# An enriched maternal environment and stereotypies of sows differentially affect the neuro-epigenome of brain regions related to emotionality in their piglets

**DOI:** 10.1080/15592294.2023.2196656

**Published:** 2023-05-16

**Authors:** Patricia Tatemoto, Fábio Pértille, Thiago Bernardino, Ricardo Zanella, Carlos Guerrero-Bosagna, Adroaldo José Zanella

**Affiliations:** a Center for Comparative Studies in Sustainability, Health and Welfare, Department of Preventive Veterinary Medicine and Animal Health, School of Veterinary Medicine and Animal Science, FMVZ, University of São Paulo, Pirassununga, São Paulo, Brazil; b Avian Behavioral Genomics and Physiology Group, IFM Biology, Linköping University, Linköping, Sweden; c Animal Biotechnology Laboratory, Animal Science Department, University of São Paulo – Luiz de Queiroz College of Agriculture (ESALQ), Piracicaba, São Paulo, Brazil; d Graduation Program in One Health, University of Santo Amaro, São Paulo Brazil; e Faculty of Agronomy and Veterinary Medicine, University of Passo Fundo, Passo Fundo, Rio Grande do Sul, Brazil; f Physiology and Environmental Toxicology Program, Department of Organismal Biology, Uppsala University, Uppsala, Sweden

**Keywords:** Maternal environment, stereotypies, prenatal, offspring, brain, emotionality, epigenetics, DNA methylation

## Abstract

Epigenetic mechanisms are important modulators of neurodevelopmental outcomes in the offspring of animals challenged during pregnancy. Pregnant sows living in a confined environment are challenged with stress and lack of stimulation which may result in the expression of stereotypies (repetitive behaviours without an apparent function). Little attention has been devoted to the postnatal effects of maternal stereotypies in the offspring. We investigated how the environment and stereotypies of pregnant sows affected the neuro-epigenome of their piglets. We focused on the amygdala, frontal cortex, and hippocampus, brain regions related to emotionality, learning, memory, and stress response. Differentially methylated regions (DMRs) were investigated in these brain regions of male piglets born from sows kept in an enriched vs a barren environment. Within the latter group of piglets, we compared the brain methylomes of piglets born from sows expressing stereotypies vs sows not expressing stereotypies. DMRs emerged in each comparison. While the epigenome of the hippocampus and frontal cortex of piglets is mainly affected by the maternal environment, the epigenome of the amygdala is mainly affected by maternal stereotypies. The molecular pathways and mechanisms triggered in the brains of piglets by maternal environment or stereotypies are different, which is reflected on the differential gene function associated to the DMRs found in each piglets’ brain region . The present study is the first to investigate the neuro-epigenomic effects of maternal enrichment in pigs’ offspring and the first to investigate the neuro-epigenomic effects of maternal stereotypies in the offspring of a mammal.

## Introduction

Epigenetic changes are major players associated with the effects of the prenatal environment on foetal programming [1]. These modifications can be maintained after mitotic events and may change in the animal in response to environmental stimuli [2]. The epigenetic modification of chromatin, including DNA methylation at CpG dinucleotides, is a key regulator of gene expression, growth, and differentiation in virtually all tissues, including brain [3,4]. Changes in DNA methylation status at specific genomic loci correlate with several traits, including social cognition [5], learning, and memory [6,7]. Furthermore, epigenetic processes are associated with dysregulated gene expression in various human psychiatric disorders [8–10], such as autism [11,12], schizophrenia [11], depression, and Alzheimer’s disease [13–15]. Besides the potential to understand the molecular aetiology of neurodevelopmental disorders in humans, epigenetic studies can also help to understand the basis of stress response in animals. Billions of animals around the world, especially those in production environments, are confined from day to day, facing multiple stressors during their lifetime. Studying the epigenetic basis of stress response in farm animals could provide important insights to develop future strategies to improve animal welfare in the production environment.

One of the most common stressors observed in captivity is low environmental complexity, in other words, a barren environment lacking stimuli, which is associated with the development and occurrence of stereotypic behaviours [16,17]. Stereotypies are defined as repetitive, invariant, and apparently functionless patterns of behaviour, which are developed or exacerbated in environments of compromised animal welfare [17–22]. Stereotypies are also expressed by individuals kept in environments with reduced stimuli, where the lack of possibilities to exercise control can cause fear or frustration [16,17,19–21,23,24]. The equivalent to stereotypic animal behaviours in humans is body-focused repetitive behaviour (BFRBs) [25]. Humans performing BFRBs generally target their own body by actions such as hair pulling (trichotillomania), skin picking, and nail biting [26]. Some BFRBs can be reduced with selective serotonin reuptake inhibitors (SSRI) [27]; however, severe manifestations are perseverating and hard to treat [28]. BFRBs associate with difficulty in managing unpleasant emotions such as boredom, anxiety, tension, and frustration and sometimes associate with emotions developing after the BFRB events, such as fear, shame, sadness, and anger [29]. Stereotypies have also been associated with repetitive and rhythmic movements in children with autism spectrum disorder and intellectual disability, with individual variation in the expression of the stereotypic behaviour [30,31]. Most of the knowledge on stereotypies associated with sensory-restricted environments in animals comes from non-human species [30]. This is because it is ethically challenging to implement balanced experimental designs using human subjects. In this context, the domestic pig (*Sus scrofa domesticus*) has proved to be an excellent model in the field of neuroscience because it has a similar brain to humans [32–34].

The expression of stereotypies has been investigated in several fields, including its genetic basis [35], personality predisposition [36,37], individual variation [37], and predisposition in relation to sex [38]. Our group has previously reported genetic mechanisms involved in different stereotypic behaviours in pigs [39]. Stereotypies may help individuals to cope with challenging circumstances [21,40,41]. However, the outcomes of employing stereotypies as a coping strategy are likely to change over time. Some studies have shown that individuals expressing stereotypies are phenotypically more plastic in being able to handle challenges than individuals not expressing stereotypies [37]. Interestingly, since stereotypies occur after long-term exposure to poor environments, there is usually no association found between this trait and high cortisol levels [19,42]. This increases the challenge of employing stereotypies as welfare indicators.

Interestingly, the expression of stereotypies can be modulated in some individuals by providing a more stimulating environment [30]. Some environmental variables have a greater impact on the occurrence of stereotypies than others [43]. Environmental enrichment involves modification of housing conditions to improve the quality of life of confined animals [44]. Enrichment exposes individuals to greater stimuli, allows them to express natural behaviours, and has been shown to reduce stereotypies [44–46]. Most of the studies investigating environmental enrichment involve applying it to the offspring whose mothers were exposed or not to stress and then assessing whether the triggered detrimental effects can be reversed after birth [47–49]. Maternal stress is shown to produce permanent and profound effects in the offspring’s brain [50]. The binding of high quantities of glucocorticoids to receptors could disrupt the development of important brain structures involved in the emotional homoeostasis of the offspring [51–53]. Recent studies have shown environmental enrichment to have a neuroprotective role in brain development and ageing [14], as well as in increasing brain plasticity [54,55]. Environmental enrichment can regulate the activity of the hypothalamic–pituitary–adrenal (HPA) axis [56–58] and reduce DNA methylation in genes normally expressed in the hippocampus and frontal cortex [59]. Epigenetic regulation has been described to be involved in stress response in all organs of the HPA axis and is suggested to modulate resilience vs vulnerability [60]. Classic experiments have shown in rats that rich maternal behaviour such grooming and licking can alter the brain epigenome of the offspring and is associated with lower corticosterone and anxiety levels [61]. In humans, environmental enrichment is starting to be applied for the recovery of human medical conditions, such as brain stroke, due to its ability to foster brain plasticity [62]. Genes affected by environmental enrichment in the brain are mainly involved in neuronal structure, synaptic signalling, and plasticity [63]. Some of these genes are also known to associate with learning and memory [64]. Moreover, enrichment affects brain weight, increases arborization and density of dendritic spines [65], and modulates neurogenesis in the hippocampus [66]. In terms of maternal enrichment, most studies focus on offspring effects triggered by maternal enrichment before reproduction [67–69]. A study performed in mice has investigated the effects of gestational maternal enrichment and finds that it influences hippocampal cell proliferation of only female foetuses and affects only female offspring in relation to locomotor activity and time spent in the centre of an open-field arena [70]. In humans, the prenatal period has been a central topic in psychiatric disorder research because several essential interactions are established during this period [50]. Despite the difficulties connecting prenatal exposures to cognitive consequences later in life, there is now convincing evidence that in utero exposure to historic famines, i.e., the Dutch Hunger Winter and the Chinese Great Leap Forward, is shown to increase the risk of long-term physical and mental detrimental consequences later in life [71]. It is important to point out that a famine involves both nutritional deficits and emotional stress. The brain epigenome is particularly affected by exposures occurring during the prenatal period [72–74]. The present study is the first to investigate the neuro-epigenomic effects of maternal enrichment in pig’s offspring, and the first to investigate the neuro-epigenomic effects of maternal stereotypies in the offspring of a mammal.

Improving the mother’s welfare during pregnancy can lead to positive changes in the offspring through foetal programming. For instance, environmental enrichment during gestation influences the activity of the HPA axis in piglets, as evidenced by reduced salivary cortisol concentration, and less nosing behaviour and aggressiveness [75]. Additionally, increasing fibre content in the diet of sows during pregnancy reduces aggressiveness in the piglets [76]. These behavioural and hormonal changes observed in response to environmental enrichment are concordant with improved piglet welfare. The maternal stereotypic behaviour of mothers also affects the emergence of stereotypies in their offspring. For example, stereotypies expressed by the mother during gestation are related to changes in piglets’ emotionality [77] and decreased fear response [78].

Because the expression of stereotypies during gestation affects emotionality in the offspring [77,78], and the fact that emotions are intrinsically related to welfare, it is worth investigating which biological mechanisms change the offspring’s phenotype. Emotions reflect animals’ ability to subjectively experience the states of the nervous system, avoid harm, and seek valuable resources or rewards [79]. Emotions also coordinate mechanisms that guide the animal to take appropriate action [80]. For instance, fear enables the individual to avoid or cope with danger [79,81]; it has a fundamental survival function and is phylogenetically maintained across many species. Welfare problems can arise if the individual has no control over the challenges it faces in its environment [18,19]. Coping mechanisms, including emotionality and motivation, are constantly affected by the environment and may, in turn, alter the epigenome. However, the extent to which stereotypic maternal behaviours affect the epigenome of brain regions involved in emotionality in the mammalian offspring is unknown.

In this study, we investigated the impact of maternal environment and stereotypies during gestation on the methylome of different brain structures involved in emotionality in piglets, namely the amygdala, frontal cortex, and hippocampus. A previous study involving the same sows employed here shows maternal stereotypy affects fear response in their piglets [77,78]. However, no molecular analyses were performed, which is investigated here. Elucidating the molecular mechanisms involved in animals’ emotions will also help us to understand the basal fundaments of human emotions and motivations [79]. In this context, epigenetic changes can uncover molecular mechanisms involved in emotional plasticity, and provide a tool to increase the welfare and resilience of humans and other animals facing stressful situations.

## Material and methods

### Animal handling and housing conditions

This study was approved by the Ethics Committee on Animal Use of the Faculty of Veterinary Medicine and Animal Science, University of São Paulo (protocol number 6157201114). The study started on a private nucleus farm located in the state of Paraná, Brazil, in which sows are maintained according to conventional practices, i.e., on concrete floors. Sixty genetically homogenous sows (TopGen Afrodite®) in the final third of gestation and housed in six pens (10 animals per pen) were divided into two treatment groups. All six pens used were conventional concrete-built structures with a concrete floor. In one group, three pens, starting from the 90th day and to the end of the gestational period 30 sows were maintained in an enriched environment (E) supplied with hay as bedding material, which was replaced daily between 08:00 h and 11:00 h. Sows in the other group (N=30), kept in three pens, were maintained in original barren environment (B) with direct contact with the concrete floor. Then, from the sows assigned to either the E or the B group, 18 animals were randomly selected per group, totaling 36 pregnant sows (six sows per pen). Then, 9 of these 18 sows per group (n=18) were assigned to the brain epigenome experiments, carried out in samples collected from their offspring. These sows were the mothers of the piglets used later in the study

Sows were offered food twice daily, at 07:00 h and 11:40 h. The food consisted of a commercial diet composed of corn, soybean meal, and a vitamin and mineral premix. All sows received the same diet for all treatments. Animals had access to water *ad libitum*. Each pen was 6 m long and 3.86 m wide, with a solid/slatted concrete floor area of 3.97 m in length and 0.85-m high walls. The E group was provided with hay in half of the pen. The feeder was 5 m long and 0.37 m wide.

Just before parturition, sows maintained after day 90 of pregnancy in both E and B environments were transferred to conventional farrowing crates and stayed there during lactation (until day 28). Therefore, piglets born from either E or B sows experienced the same environment from birth to the end of the experiment. At birth, the umbilical cord of each piglet was tied with a string previously immersed in an antiseptic solution and dipped in iodine (10%) for 5 seconds. Piglets were then cleaned using paper towels and assigned a number reflecting their birth order on the back using a non-toxic marker. After this initial standard management procedure, piglets were placed with their mother to ingest colostrum. On the first day of life, piglets’ teeth were ground, their tails were docked, ears were notched, and individual weight was recorded, in line with the farm’s routine practice.

Piglets were weaned at 28 d of age, vaccinated (against *Porcine circovirus*, *Streptococcus suis*, *Haemophilus parasuis*, and *Mycoplasma hyopneumoniae*), and transported from the farm in Jaguariaíva, Paraná (where the first stage of the experiment was carried out) to the Fernando Costa Campus of the University of São Paulo in Pirassununga, São Paulo state. The journey was approximately 8 hours in duration. From each of the 36 pre-selected sows, one pair of piglets was assessed for the second part of the experiment (*N* = 72 piglets). During transportation, two litter-mates were placed in a box (73.5 cm long, 53 cm wide, 21 cm high) bedded with hay. After weaning, the 72 animals were kept in conventional suspended nursery pens, with six litters kept in the same pen. Each pen had 12 animals, a pair from each sow, grouped according to their treatment during gestation (E or B sows). Piglets had *ad libitum* access to water and a commercial pig diet. Brain tissue samples were collected from one male piglet randomly selected per sow, as they were slaughtered, while female piglets were kept in the farm as sow replacements. Euthanasia was performed using a captive bolt as a stunning method (Accles & Shelvoke, Dispatch Kit. 25). Immediately after stunning, all animals were subject to exsanguination. This method is recommended and accepted by Brazilian legislation (CONCEA and CFMV resolution 1000) and considered as humane by the AVMA Guidelines for Humane Slaughter of Animals (2016) and the American Meat Institute Guidelines (2013, p. 19–20). Dissections of brain regions were carried out using standard operating procedures in our laboratory, adapted from Fleming et al. (2021) [82].

### Experimental design

**Figure 1.**
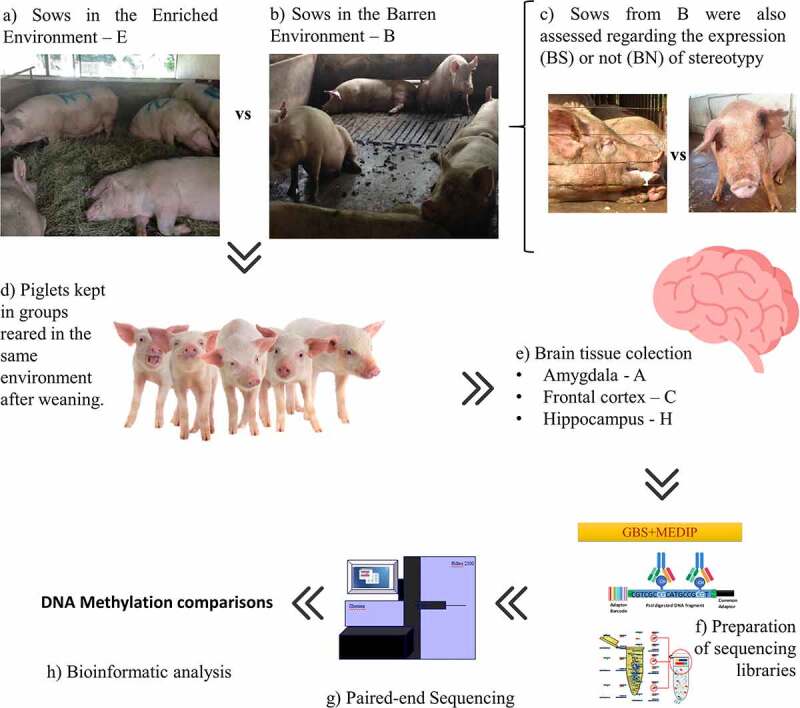
Experimental design. Sows were maintained in an enriched (a) or barren environment (b). Sows kept in the barren environment were subdivided into two groups (c) depending on whether they expressed (BS) or did not express stereotypy (BN). Piglets from different prenatal environments were kept in the same conditions after weaning, with no difference between the pens (d). Males were slaughtered 35 d after weaning and the brain tissues collected (e). Finally, the DNA was extracted and GBS-MEDIP (genotyping by sequencing combined with methylated DNA immunoprecipitation) sequencing libraries were prepared for (g) paired-end sequencing. The sequences of the reduced methylomic fractions of individuals were then bioinformatically analyzed (h).

To measure the epigenetic effects of the prenatal environment, we analysed the methylomes of brain tissues of weaned piglets; namely the amygdala (A), frontal cortex (C), and hippocampus (H). We compared male piglets born from sows kept in the enriched environment (E; *N* = 9) with piglets born from sows kept in the barren environment (B; *N* = 9). Then, within the B group, we compared the brain methylomes of piglets born from sows expressing stereotypic behaviour (BS; *N* = 5) with those from sows not expressing stereotypic behaviour (BN; *N* = 4) and with those from sows in the E group (*N* = 9). For the contrasts involving stereotypies, we only used brain tissue from piglets from sows expressing stereotypies within the barren group (BS) because the vast majority of sows expressing stereotypic behaviour in the E group ceased the behaviour after the enrichment started on day 90 (6 out of 7 sows ceased the behaviour), despite the fact that the number of sows expressing stereotypies is (expectedly) equivalent among the experimental groups before day 90 (Supplementary Spreadsheet S1). Conversely, in the B group, all sows expressing stereotypies before day 90 of pregnancy maintained the behaviour afterwards (5 out of 5 sows). The criterion to define a sow as expressing stereotypic behaviour across the experiment was that a sow expressed stereotypy at least one time before and one time after day 90, when the enrichment started in group E. Piglets were assigned to the BS group (*N* = 5) if their mothers were classified as expressing stereotypies across the experiment. Piglets from the BN group (*N* = 4) were those from sows who never displayed stereotypies prior to data collection. From the initial cohort of 72 piglets, 18 were randomly assessed according to two criteria observed in their mothers: i) how they were raised (E or B) and ii) whether they expressed stereotypic behaviour or not within the B group (BS or BN). From here, the piglets will be referred to in relation to their mothers’ environments, E or B. The workflow of the experimental design can be seen in Figure 1.

### Brain tissue collection and DNA extraction

Transportation, stunning, and slaughter were monitored and controlled to ensure good welfare practices and to minimize the impact of the procedures on male piglet brain collection. The brain of each animal was weighted and dissected. Brain tissues from the A, C, and H of each animal were collected and immediately frozen in liquid nitrogen.

DNA extraction from the dissected brain tissue samples was performed with the Invitrogen® PureLink Genomic DNA Mini kit, following the manufacturer’s instructions. Thirty grams of each dissected brain tissue was incubated with proteinase K overnight at 56ºC. An automatic system allowed homogenization of these solutions every 30 minutes to lyse the tissue cells. After extraction, DNA was eluted in 100 μl of elution buffer, and a NanoDrop system was used to assess the purity and amount of the DNA based on 260/280 and 260/230 ratios.

### Sequencing library preparation

To analyse DNA methylation in the reduced genomic fractions of many individuals, we combined genotyping by sequencing (GBS) [83] with methylated DNA immunoprecipitation (MeDIP) [84], as previously described [85]. Briefly, the individual DNA samples are first digested with the *Pst*I (ThermoFisher Scientific) restriction enzyme, whose recognition site does not contain CpGs. Then, Illumina adapters and individual barcodes were added to the fragments produced after this digestion of individual DNA samples [86] to later identify individual fragment samples bioinformatically [86,87]. The individually barcoded samples are then pooled and subsequently subjected to enrichment of the methylated fraction (MeDIP) by an anti-methyl-cytosine antibody (2 μg/μl; catalogue number C15200006; Diagenode, Denville, NJ, USA), as previously described [84]. PCR was then performed after the MeDIP capture of the methylated fraction of the pool of barcoded DNA samples to create the sequencing library [85]. Paired-end sequencing was performed with a read length of 100 bp on the Illumina HiSeq2500 platform, at the Animal Biotechnology Laboratory (ESALQ/USP), Brazil.

### Bioinformatic analyses

Stacks v.1.39 was used for data de-multiplexing [88] and for quality trimming of the reads, using default parameters. In this procedure, each read stored in a FASTQ file has an identification map key file; a barcode containing matching information for the respective sample. Expected reads begin with one of the individual barcodes and are followed by the cut site remnant for *Pst*I, which contains the sequence 5´ CTGCA 3´. Fragments are then grouped into individual files, which correspond to individuals identified by their respective barcodes.

The option ‘very sensitive-local alignment’ was used in the Bowtie2 tool v.2.2.5 [89] to align quality-trimmed reads against the pig reference genome (*Sus scrofa* 11.1, NCBI). Default parameters for paired-end sequences were used. The coverage depth of each sample was checked using Samtools v.0.1.19 [90] with the ‘depth’ option.

Our method for identifying significant differentially methylated regions (DMRs) employs two statistical approaches. First, following read alignment, we merged sequences of animals from each group to identify peaks of sequencing coverage between treated and controls using the MACS2 (v.2.1.1) program with default parameters. This program generates a .bed file with the positions of each peak (passed false discovery rate [FDR] ≤ 0.1 threshold criteria), which we called ‘Regions of Interest’ (ROI) to serve as input in the nearby analyses.

The second statistical approach for the identification of DMRs involves analyses performed using the following bioinformatic packages from the ‘R’ Bioconductor repository. The BSgenome.Sscrofa.UCSC.susScr11 package was uploaded as the reference genome. The MEDIPS R-package was used for basic data processing, quality controls, normalization, and identification of differentially methylated regions (DMRs). To avoid possible artefacts caused by PCR amplification, MEDIPS allows a maximum number of stacked reads per genomic position. This is done using a Poisson distribution of genome-wide stacked reads. The default parameter of *p* = 0.001 was used as the threshold to detect stacked reads. MeDIP-seq data were transformed into genome-wide relative methylation scores using a CpG-dependent normalization method [91]. This normalization is based on the dependency between short-read coverage and CpG density at genome-wide windows [92] and can be visualized as a calibration plot. A calibration plot was generated using one of the individuals from each test to generate a coupling set (an object that groups genome-wide information about CpG density) to each specific treated vs. control test. Based on this, a threshold for a minimum sum of counts across all samples per window was defined (minRowSum = 10; meaning 10 counts per ROI).

Sequencing data for each individual were then assigned to one of the experimental groups. Differential coverage (i.e., differential methylation) was calculated between the two pre-defined conditions. ROIs were considered DMRs after passing the threshold of *p* ≤ 0.05. DMRs passing this threshold were used for exploratory analysis of gene-related enrichment pathways. Additionally, DMRs that passed the threshold of FDR ≤ 0.6 (adjusted) were considered of special interest and used to identify relevant genes.

All DMRs identified in the analysis were then annotated against the pig reference genome (*Sus scrofa* 11.1; NCBI) using the R packages described below to obtain the location of each DMR in relation to their related genes and to obtain information about the distance between each DMR and the nearest transcription start site (TSS). First, we extracted the coordinates from each DMR and from the annotated genes of the pig genome (using the org.Ss.eg.db package). Then, we overlapped the identified DMR with these annotated genes using the Genomic Ranges R package. Next, we performed functional genomic annotation of the DMR overlapping with genes. For this, we used the annotatePeak function in the ChIPseeker package with default parameters [93], which are defined as follows: the promoter is defined as the 3kbps before the TSS of an annotated gene, while the downstream region is defined as the 300 bps after the TTS of the gene. The annotatePeak function also assigns intergenic DMRs to the nearest annotated gene. In this function, we forged a ss_txdb object using the GenomicFeatures and org.Ss.eg.db packages. The latter is the functional annotation database for the pig genome (BSgenome.Sscrofa.UCSC.susScr11). The ss_txdb for *Sus scrofa* was extracted from the transcript metadata TxDB, which contains all the functional annotations available at the UCSC Genome Browser. For this, we used the function makeTxDBFromUCSC (using the parameter: genome = ‘susScr11’).

After discovering specific features related to the identified DMRs, we checked their distribution in relation to the TSS of the nearest gene. These distances were categorized in ten, hundred, thousand, and million numerical magnitudes. Analysis includes every distance that was counted at least once.

Overlap analysis to identify DMRs obtained was performed based on permutation tests (*N* = 100), which determined whether peak overlaps were significant. For this, we used the findOverlapsOfPeaks function from the ChIPpeakAnno v3.6.5 R package with default parameters. Venn diagrams were plotted using the *makeVennDiagram* function within the same package.

To describe terms related to molecular functions, cellular components, and biological processes from each gene analysed in this study, we used the enrichGO function of the Gene Ontology (GO) database. This approach aims to find the best-clustered, predefined, gene-related groups using the compareCluster function from the package ChIPseeker [93].

To better visualize the different biological functions affected by the genes associated with our data, we built graphical representations of the main pathways affected per contrast across all the tissues analysed, as well as how they interconnect and relate to genes using the GOnet [94] webtool (http://tools.dice-database.org/GOnet/).

## Results

### Raw data analysis

Methylation analysis was carried out using a linear model with 12 different contrasts (Table 1) including the environment to which the sows were exposed during gestation (E or B), whether or not they expressed stereotypies (BN or BS), and the three brain structures examined.

**Table 1.** Twelve distinct contrasts used to compare DNA methylation between the groups, four for each brain tissue.

Tissue	Group 1	Group 2	Contrast
A	E	B	Methylation differences in the amygdala (A) from the offspring of sows reared in an enriched environment (E) compared with those reared in a barren environment (B)
A	E	BS	Methylation differences in the amygdala (A) from the offspring of sows reared in the enriched environment (E) compared with those reared in the barren environment and expressing stereotypies (BS)
A	E	BN	Methylation differences in the amygdala (A) from the offspring of sows reared in the enriched environment (E) compared with those reared in the barren environment not expressing stereotypies (BN)
A	BS	BN	Methylation differences in the amygdala (A) from the offspring of sows reared in the barren environment and expressing stereotypies (BS) compared with those in the barren environment not expressing stereotypies (BN)
C	E	B	Methylation differences in the frontal cortex (C) from the offspring of sows reared in the enriched environment (E) compared with those reared in the barren environment (B)
C	E	BS	Methylation differences in the frontal cortex (C) from the offspring of sows reared in the enriched environment (E) compared with those reared in the barren environment and expressing stereotypies (BS)
C	E	BN	Methylation differences in the frontal cortex (C) from the offspring of sows reared in the enriched environment (EE) compared with those reared in the barren environment and not expressing stereotypies (BN)
C	BS	BN	Methylation differences in the frontal cortex (C) from the offspring of sows reared in the barren environment and expressing stereotypies (BS) compared with those not expressing stereotypies (BN)
H	E	B	Methylation differences in the hippocampus (H) from the offspring of sows reared in the enriched environment (E) compared with those reared in the barren environment (B)
H	E	BS	Methylation differences in the hippocampus (H) from the offspring of sows reared in the enriched environment (E) compared with those reared in the barren environment and expressing stereotypies (BS)
H	E	BN	Methylation differences in the hippocampus (H) from the offspring of sows reared in the enriched environment (E) compared with those reared in the barren environment and not expressing stereotypies (BN)
H	S	N	Methylation differences in the hippocampus from the offspring of sows reared in the barren environment and expressing stereotypies (S) compared with those not expressing stereotypies (N)

The average sequencing and alignment statistics for the piglets used in this study are shown in Supplementary Spreadsheet S2.

### Library size

*In silico* digestion was performed to validate the use of the restriction enzyme *Pst*I for the library preparation in pigs (BSgenome.Sscrofa.UCSC.susScr11). A representation of the expected results if all CpG sites in the entire pig genome (after cleavage and immunoprecipitation steps) were methylated can be found in Supplementary Figure S1. The in-silico digestion produced fragmented DNA at the size 200–500 bp range, which is suitable for the downstream steps of the protocol and Illumina sequencing (Supplementary Figure S1).

### CpG enrichment

The enrichment score of methylated CpGs in the genomic regions covered by the set of reads sequenced was calculated in relation to the reference genome (*Sscrofa11.1, INSDC Assembly GCA_000003025.6, Dec 2016*) using the MeDIPS package [95]. We identified an ‘enrichment score’ of 2.75 (±0.06) and, on average, 112,000 CpGs were covered (Supplementary Spreadsheet S2), corresponding to 0.38% of the CpG regions in the *Sus scrofa* genome.

### Analysis of differentially methylated regions (DMRs)

We use a two-stage statistical analysis to pinpoint significant DMRs. First, from differential coverage peaks called in the comparison performed in each contrast, we obtained regions of interest (ROI) defined by an FDR-adjusted *p* ≤ 0.1 in MACS2 peak calling. Next, we conducted a second statistical test using EdgeR statistics (within the MeDIPs R package) to make a final selection of significant DMRs. From this step, we extracted two sets of DMRs. The first set involved those DMRs passing an unadjusted p-value≤0.05, which were employed for exploratory analysis of enrichment pathways based on DMR-related genes, and to identify overlapping DMRs and DMR-related genes among the contrasts used. Additionally, those DMRs passing the cut-off threshold of false discovery rate (FDR) adjusted *p* ≤ 0.6, called top-DMRs, were used for a detailed description of the genes related to significant DMRs. Table 2 shows the number of ROIs and DMRs identified for each contrast used in this study.

**Table 2.** Total number of genomic windows sequenced and tested in the three brain structures, plus the number of DMRs identified using different *p*-value thresholds. The brain structure is signalized by the first letter of the treatment column, in which ‘C’ is the frontal cortex, ‘A’ is the amygdala, and ‘H’ is the hippocampus.

Contrast	minRowSum = 10	ROI ≤ 0.1	DMR *p* ≤ 0.05	DMR FDR ≤ 0.6
A-E/B	136,522	3,969	80	0
A-E/BS	154,422	2,463	57	0
A-E/BN	51,298	1,376	33	0
A-BS/BN	19,179	470	26	0
C-E/B	143,704	3,919	122	0
C-E/BS	47,831	3,289	77	2
C-E/BN	51,392	2,115	50	1
C-BS/BN	16,139	409	13	0
H-E/B	178,305	6,559	177	2
H-E/BS	179,640	4,283	119	3
H-E/BN	190,455	4,158	97	4
H-BS/BN	19,759	808	19	0

**Figure 2.**
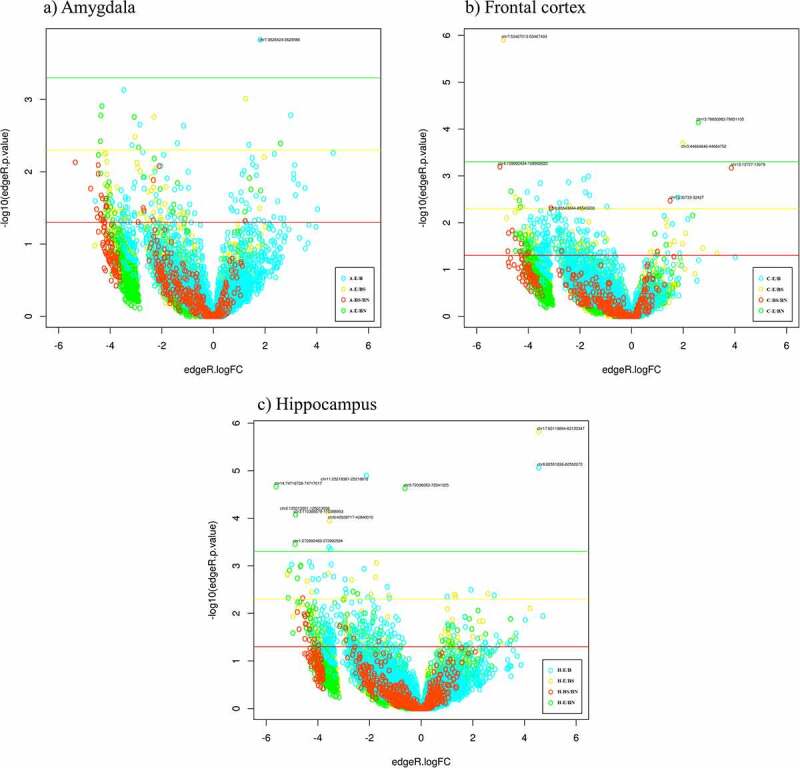
**Volcano plots representing the *p*-values (–log10; y axis) and fold changes (x axis) of the DMR obtained with the four contrasts employed for each piglet brain structure (a, b, and c)**. The thresholds represented by coloured lines, correspond to the *p*-values 0.05 (red), 0.005 (yellow), and 0.0005 (green). DMRs passing the FDR ≤ 0.6 threshold are labelled in the figure.

Volcano plots were used to visualize the distribution of combined ROIs obtained with all the contrasts pertaining to each tissue in relation to the *p*-values (y-axis) and fold changes (x-axis) (Figure 2). The hippocampus had the highest number of DMRs passing the most stringent *p* value (p**≤**0.0005).

**Figure 3.**
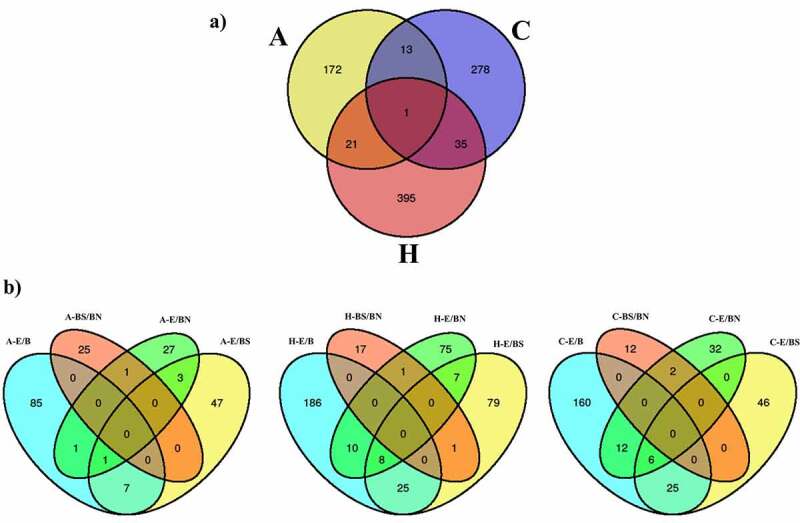
Venn diagrams showing (a) combined DMRs identified per tissue and (b) DMRs identified per contrast in each tissue: amygdala (A), frontal cortex (C) and hippocampus (H).

The DMRs identified from the studied contrasts were visualized using Venn diagrams (Figure 3). When combining all the contrast performed per tissue, it is observed that the hippocampus has the highest number of DMRs, followed by the frontal cortex and the amygdala. Most of these DMRs are unique for each tissue. Of all the contrasts, the single brain tissue with the highest number of unique DMRs was the hippocampus (*N* = 395), followed by the frontal cortex (*N* = 278) and amygdala (*N* = 172). Considering the E/BS comparisons across tissues, the hippocampus had the highest number of unique DMRs (*N* = 79), followed by the amygdala (*N* = 47) and frontal cortex (*N* = 46). Considering the E/BN comparisons across tissues, the hippocampus also had the highest number of unique DMRs (*N* = 75), followed by the frontal cortex (*N* = 32) and amygdala (*N* = 27). Finally, considering the BS/BN comparisons, the amygdala had the highest number of unique DMRs (*N* = 25), followed by the hippocampus (*N* = 17) and frontal cortex (*N* = 12).

The hippocampus and frontal cortex (*N* = 36) had the highest number of DMRs in common, followed by the amygdala and hippocampus (*N* = 22), and by the amygdala and frontal cortex (*N* = 14) (Figure 3a). In addition, there was one region of the pig genome (chr7: 3628367–3628578) containing nine DMRs overlapping all the analysed tissues (Figure 3a). This region emerged from all the contrast related to the frontal cortex plus the contrasts H-E/BN, H-E/B, H-E/BS, A-E/B, and A-BS/BN. This region is located less than 3 kb distal to the gene *NRN1* (Table 3). When combining the DMRs obtained from all the contrasts within each tissue (Figure 3b), it is observed that, across tissues, most DNA methylation differences emerge within the E/B contracts. Also, most DNA methylation differences in BS/BN are observed in the amygdala. Interestingly, the DMRs emerging from the BS/BN contrasts do not overlap with the DMRs emerging from the E/B contrasts. Also, the largest number of overlapping DMRs between two contrasts was observed between the E/B and the E/BS contrasts and between the E/B and the E/BN contrasts. These trends are more prominent in the hippocampus and frontal cortex than in the amygdala. It is also notorious that the DMRs related to the BS/BN contrasts present minimum overlaps with the other contracts in every tissue. No DMR overlaps all contrasts in each particular tissue.

**Table 3.** Genomic features and annotation of DMRs with the lowest p-values (FDR ≤ 0.6).

Tissue	Contrast and hypermethylation direction	Location	p-value	FDR.p.value	Annotation	Ensembl ID	Symbol	Description	Source
A	E > B	chr7:3,628,424–3,628,586	1.48E–04	0.589	Distal Intergenic	ENSSSCG00000026211	NRN1	Neuritin 1	NCBI gene; Acc:100155948
C	BS > E	chr3:44,664,646–44,664,752	1.99E–04	0.327	Distal Intergenic	ENSSSCG00000043043	-	-	-
C	BS > E	chr7:534,670,13–53,467,493	1.24E–06	0.004	Intron	ENSSSCG00000001818	FES	FES proto-oncogene, tyrosine kinase	NCBI gene; Acc:100155631
C	BN > BS	chr6:85,543,044–85,543,203	4.88E–03	0.499	Promoter (2-3kb)	ENSSSCG00000029097	RCC1	Regulator of chromosome condensation 1	NCBI gene; Acc:100621543
C	BN > BS	chr15:13,727–13,979	6.73E–04	0.138	Distal Intergenic	ENSSSCG00000047217	-	-	-
C	BN > BS	chr15:30,733–32,427	3.39E–03	0.463	Distal Intergenic	ENSSSCG00000047217	-	-	-
C	BN > E	chr13:78,850,983–78,851,105	7.25E–05	0.153	Distal Intergenic	ENSSSCG00000040341	U6	U6 spliceosomal RNA	RFAM; Acc:RF00026
H	B > E	chr8:82,551,826–82,552,070	8.59E–06	0.042	Distal Intergenic	ENSSSCG00000047140	-	-	-
H	E > B	chr11:25,218,381–25,218,612	1.28E–05	0.042	Intron	ENSSSCG00000029837	VWA8	von Willebrand factor A domain containing 8	NCBI gene; Acc:100511470
H	E > BS	chr2:125,013,301–125,013,556	7.26E–05	0.155	Intron	ENSSSCG00000042728	U6	U6 spliceosomal RNA	RFAM; Acc:RF00026
H	E > BS	chr6:42,839,717–42,840,010	1.12E–04	0.159	Intron	ENSSSCG00000040905	FAAP24	FA core complex associated protein 24	NCBI gene; Acc:100621643
H	BS > E	chr17:63,119,894–63,120,347	1.52E–06	0.006	Distal Intergenic	ENSSSCG00000043535	-	-	-
H	E > BN	chr1:272,992,483–272,992,594	3.50E–04	0.364	Intron	ENSSSCG00000031407	STKLD1	Serine/threonine kinase like domain containing 1	NCBI gene; Acc:110256522
H	E > BN	chr3:110,388,579–110,388,953	8.43E–05	0.117	Intron	ENSSSCG00000030147	TRMT61B	tRNA methyltransferase 61B	NCBI gene; Acc:100520017
H	E > BN	chr5:72,036,053–72,041,325	2.35E–05	0.049	Distal Intergenic	ENSSSCG00000041614	-	-	-
H	E > BN	chr14:74,716,726–74,717,017	2.16E–05	0.049	Intron	ENSSSCG00000010280	VSIR	V-set immunoregulatory receptor	NCBI gene; Acc:100154373

**Figure 4.**
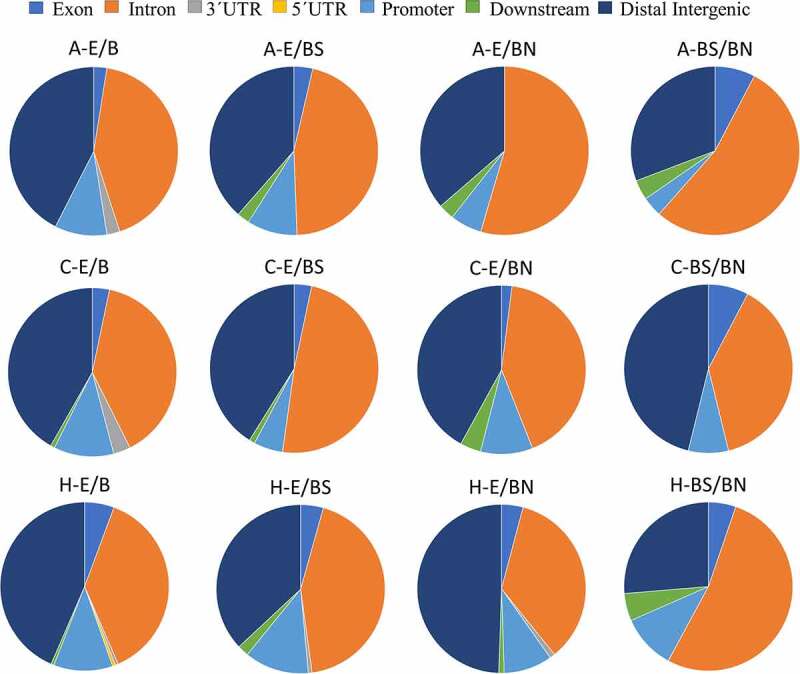
Pie charts showing the genomic location of DMRs found in relation to each brain tissue and experimental contrast.

Functional annotation of the significant DMRs showed that the distribution patterns of DMR locations in relation to genes (Figure 4) were similar among the brain tissues analysed (A, C, and H). Among the contrasts, the distribution patterns of DMR locations in relation to genes were very similar between the treatments E and B. However, genomic patterns of functional annotations differ between the contrasts E/BN and BS/BN. For these two contrasts, the amygdala had the highest proportion of DMRs in intronic regions, but the lowest proportion of DMRs in promoters compared to the other tissues. In the contrasts A-BS/BN, C-BS/BN, and H-BS/BN, the amygdala and hippocampus showed similar patterns, while the frontal cortex showed a lower proportion of DMRs in the intronic regions and a higher proportion in the distal intergenic region.

We also calculated the distances between each significant DMR and their nearest gene TSS by using all the combined DMRs obtained per tissue (Supplementary Figure S2). The peaks of DMRs described next are described as distances to the nearest TSS. For the E/B contrasts we observed peaks at −20 Kbps, −10 Kbps, +20 Kbps, and+30 Kbps in the hippocampus, and at −10 Kbps, and+20 Kbps in the frontal cortex. There were no peaks of equivalent magnitude in the amygdala. For the E/BS contrasts, the identified peaks were located at −10 Kbps, and+20 Kbps in the hippocampus and at +20 Kbps in the frontal cortex. There were no peaks in the amygdala. For the E/BN contrasts, we observed peaks at −10 Kbps, +20 Kbps, and+50 Kbps in the hippocampus, while in the frontal cortex the peak was located at a distance of −10 Kbps from TSS. There were no peaks in the amygdala. For the BS/BN contrasts, there were peaks at −10Kbps, +20 Kbps, and +70Kbps observed in the amygdala and over-represented compared to the other contrasts, with peaks at +30 Kbps being evident in the hippocampus. There were no peaks in the frontal cortex. Generally speaking, peaks at  +20 Kbps were the most frequent when analysing the frontal cortex, while peaks at −10 Kbps were the most frequent when analysing the hippocampus. In the amygdala, however, peaks were only observed when comparing piglets from mothers expressing/not expressing stereotypy, with no peaks observed in the frontal cortex in this scenario.

### Analysis of DMR-related genes

Next, we investigated the genes related to the DMRs identified in each scenario. The 16 top-DMRs identified in our study were mapped to different regions of nine known genes with eight top-DMRs located in distal intergenic regions, seven in intronic regions, and one in a promoter region (2–3 kb) (Table 3; FDR ≤ 0.6). In terms of the different brain tissues, nine top-DMRs were identified with the contrasts involving the hippocampus, six with the contrasts involving the frontal cortex, and only one DMR was found with the contrasts involving the amygdala. One gene, the *U6* small nuclear RNA, was affected by top-DMRs in more than one contrast and tissues, with an intronic DMR emerging in the H-E/BS contrast and a distal DMR emerging in the C-E/BN contrast. The hippocampus had the highest number of top-DMRs (*N* = 9).

**Figure 5.**
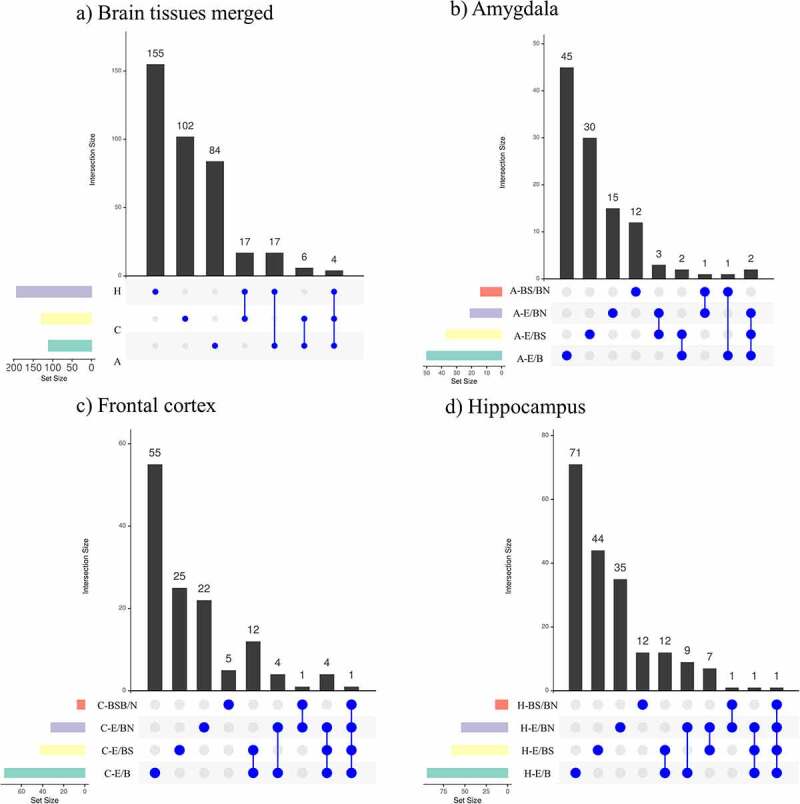
DMR-related gene overlaps obtained across contrasts in the brain structures investigated. (a) shows the DMR-related genes obtained in all tissues merged and the number of unique DMR-related genes per tissue; (b-c) show the DMR-related genes obtained in each tissue analysed (amygdala (b), frontal cortex (c), and hippocampus (d)) and the unique DMR-related genes obtained per contrast employed.

We then analysed all DMR-related genes to identify overlaps among contrasts. Figure 5 depicts unique and overlapping DMR-related genes in all tissues merged (Figure 5a) and per tissue (Figures 5b-d). The hippocampus had the highest number of unique DMR-related genes (*N* = 162) across all contrasts, followed by the frontal cortex (*N* = 107) and amygdala (*N* = 102) (Figure 5a). Four DMR-related genes overlapped among all three tissues investigated. The gene *LRATD2* (*LRAT domain containing 2; Chr4*) was related to DMRs identified with the contrasts A-E/B, C-E/B, C-E/BS, H-E/B, H-E/BN. The gene *MROH9* (*maestro heat-like repeat family member 9; Chr9*) was related to DMRs identified with the contrasts A-E/B, A-E/BS, C-E/BS, and H-E/BS. The gene *NRN1* (neuritin 1; Chr7) was related to DMRs identified with the contrasts A-E/B, C-E/B, C-E/BS, C-E/BN, H-E/B, H-E/BN. The DMRs associated to these three genes were located in distal intergenic regions. Strikingly, the gene *U6* (U6 spliceosomal RNA; Chr2) contained overlapping DMRs that were observed in 16 different overlap tests performed among the contrasts.

Across all the tissues analysed, the E/B contrasts had the highest number of unique DMR-related genes, followed by the E/BS, E/BN, and BS/BN contrasts, respectively. In terms of overlapping DMR-related genes, while the prefrontal cortex and the hippocampus each presented one DMR-related gene overlapping all contrasts, the amygdala presented none (Figure 5b,c).

### Pathway enrichment

**Figure 6.**
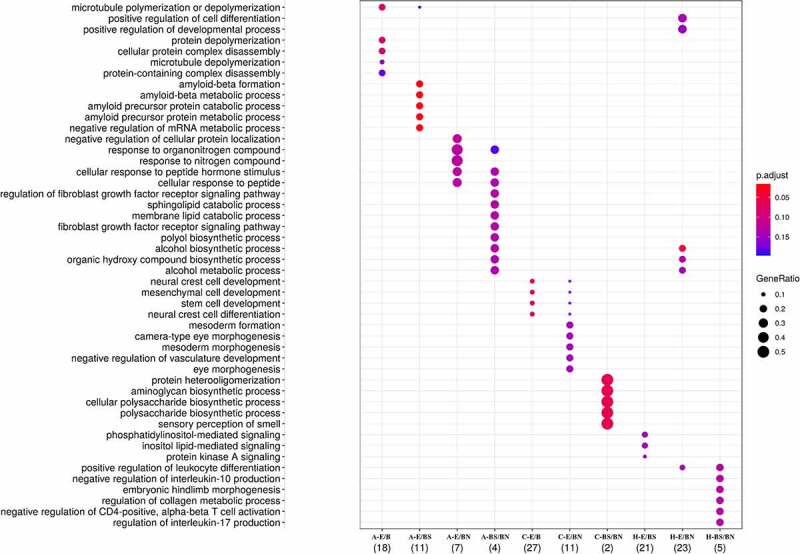
Pathways enriched with DMR-related genes obtained from each contrast employed. The depicted pathways represent those passing FDR ≤ 0.1 threshold.

GO enrichment analysis was performed to identify biological processes affected by the DMR-related genes. Interestingly, clusters of biological processes could be identified in relation to the DMR-related genes observed in each contrast (Figure 6). Interestingly, the highest number of genes in significantly enriched pathways was present in the E/B contrast in the frontal cortex (N = 27), related to neural crest development, followed by E/BN in the hippocampus (N = 23), with effects mainly on alcohol metabolism, by E/BS in the hippocampus (N = 21), with effects on lipid mediated signalling, and by E/B in the amygdala (N = 18), with effects on microtubule poly/depolymerization. Another contrast worth mentioning is E/BS in the amygdala, in which 11 genes were found in highly significant enriched pathways related to amyloid metabolic processes (Figure 6). Details of the gene enrichment analysis, such as *p* values for each pathway, can be found in Supplementary Spreadsheet S3.

**Figure 7.**
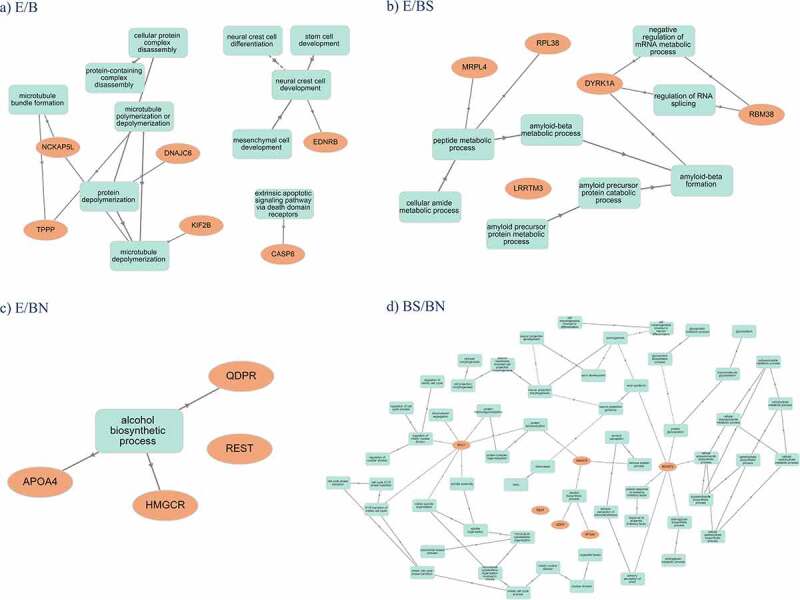
Representations of the main pathways affected per constrast across analyzed tissues and the interconnections related to genes.

Graphical representations of the main pathways affected per contrast across all the tissues analysed as well as how they interconnect and relate to genes are shown in Figure 7a-d. It is observed that the pathways emerging from DMR-related genes found in the E/B contrasts relate, in general, to microtubule assembly and neural crest development (Figure 7a), the pathways emerging in the E/BS (Figure 7b) are related to amyloid and metabolic processes, while E/BN was related to the alcohol biosynthetic process. The pathways emerging in the BS/BN contrasts related to cell cycle and neuron morphogenesis/projection are presented in Figure 7d.

## Discussion

In this study, we investigated how stereotypies of gestating sows and environmental enrichment in the final trimester of gestation can affect the neuro-epigenome of their male offspring. Previously, we have shown that both stereotypies and environmental enrichment affect the welfare of pregnant sows and their offspring [75,77,78].

In humans, stereotypies are associated with several psychiatric disorders. A recent review by Keller et al. (2021) [96] offers very strong arguments to further explore animal models relevant to this condition and encourages mapping the neurocircuitry associated with stereotypies. The present paper investigates how the maternal environment of pregnant sows and the occurrence of stereotypies in these sows are associated with methylation alterations in brain regions of relevance for emotionality in the offspring.

DNA methylation comparison involved 12 different contrasts (Table 1) including the environment to which the sows were exposed during gestation, whether they expressed stereotypies, and the three brain structures examined namely the amygdala, hippocampus, and frontal cortex. We observed methylation differences between contrasts despite the small number of animals employed in the study, which relates to low statistical power to detect differences. Thus, employing a larger number of individuals would probably uncover additional differences. Interestingly, the hippocampus, an area of the brain involved in learning, memory, and emotional regulation, presented the highest number of DMRs, top-DMRs, and unique DMRs. Additionally, the hippocampus is the most affected brain region in the three contrasts comparing an enriched vs barren maternal environment: E/B, E/BS, and E/BN, supporting its well-documented plasticity. On the other hand, despite the fact that the amygdala is the brain region with the lowest number of combined DMRs, it is the region with the highest number of DMRs (and unique DMRs) emerging in the contrast BS/NS, which compares expression vs non-expression of maternal stereotypy. These findings suggest that while the hippocampus of piglets is more sensitive to maternal environment (e.g., enrichment), the amygdala is the brain region that would primarily respond to the expression of maternal stereotypy. The hippocampus is a plastic brain region associated with learning and short-term memory, susceptible to damage by environmental stimuli, and frequently affected by neurologic and psychiatric disorders [97]. Although the hippocampal effects of maternal enrichment on the offspring have received limited attention in mammals, experiments in rats and sheep shed light on the effects of maternal behaviour and stress on the hippocampal function of their offspring. For example, adult rats born to mothers providing low levels of licking and grooming showed impaired hippocampal-dependent memory [98]. Maternal effects on the hippocampus of the offspring seem to be defined during early development. For example, sheep embryos harvested from ewes frequently separated from their flocks during early gestation (1^st^ and 2^nd^ trimesters) showed a reduced amount of neuronal processes and synaptic density both in the hippocampus and cerebral cortex [99]. Based on our results, maternal enrichment would preferentially affect the hippocampus over other tissues, raising the possibility that maternal enrichment may influence hippocampus-related functions such as short-term memory and learning. One possible mechanism is the mitigating effect of environmental enrichment on the release of glucocorticoids as the hippocampus is the area in the brain with the highest concentration of glucocorticoid receptors [100].

The amygdala, in turn, is known for its role in threat detection, fear response, and memory related to emotions [101]. In pigs, maternal stress produced by social mixing with unfamiliar conspecifics during gestation (which alters dominance hierarchy, often resulting in vigorous fighting [102,103]) is shown to produce long-lasting effects in the offspring, including increased expression of the corticotropin releasing hormone (CRH) mRNA in the amygdala [104]. Also, in pigs, pregnant sows maintained in a behaviour restrictive environment produce adult female offspring with altered CRH receptor 1 and 2 ratios in the amygdala, which indicates neurobiological propensity for anxiety-related behaviour [53]. These studies show that maternal stress and behaviour during gestation have consequences in the function of the amygdala of their offspring, with potential associated behavioural effects. Another important observation is that across tissues the DMRs emerging from the BS/BN contrasts do not overlap with the DMRs emerging from the E/B contrasts. It is worth to point out that the BS/BN contrast allows us to understand the effects of stereotypy on top of those produced by the barren environment. This is not possible in the E group, where the vast majority of the sows expressing stereotypy ceased their expression after the enrichment started. This shows that DNA methylation changes in piglets’ brains in relation to maternal stereotypy are unique and independent of those emerging due to the maternal barren environment, suggesting separate molecular pathways being influenced by these two different maternal stimuli. We also observed a high overlap between DMRs emerging from the E/B and E/BS contrasts across tissues, to a less degree in the amygdala, and from the E/B and E/BN contrasts in the hippocampus and frontal cortex. Although it is expected that overlaps are found between the E/B and E/BS or E/BN contrasts, because BN and BS are subgroups of B, it is intriguing that less overlaps are found in the amygdala, especially between the E/B and E/BN contrasts. This suggests that, compared to maternal environment, maternal stereotypy affects the amygdala of piglets in a unique and independent manner when considering all the three brain regions investigated. Based on this, maternal stereotypy would preferentially affect the amygdala, suggesting that the behavioural effects in the offspring are related to amygdala-related functions such as fear and stress response and emotional memory. There is growing interest in the role of the amygdala in empathy and social behaviour. An experimental model in rats that tested the impact of inflammatory challenge in autism-like behaviour in the offspring demonstrated a large effect and a significantly higher number of c-fos labelled cells in the basomedial amygdala (BMA) and basolateral amygdala (BLA), associated with compromised social behaviour [105].

When inquiring into the genomic functions of the identified DMRs, we observed that in E/B contrasts most DMRs were equivalently located in intronic and distal intergenic regions across tissues. Additionally, very few DMRs are located downstream of genes. This pattern changes in the BS/BN contrasts, where in the amygdala and the hippocampus, DMRs in introns outnumber DMRs in other locations. Additionally, these tissues present DMRs mostly downstream to genes to an observable fraction. These findings, together with the general observation that DMRs found here do not have a major presence in promoter regions, raise questions about the molecular mechanisms and genetic function responding to maternal experiences in mammalian embryos. DNA methylation effects on gene transcription depend on the location of the affected CpGs relative to a gene. Generally speaking, DNA hypomethylation of distal intergenic regions in vertebrates is correlated with enhancer activity [106], while DNA methylation in introns is generally associated with increased expression [107]. Additionally, it is well known that hypermethylation in promoter regions is generally associated with the downregulation or silencing of genes [108–110]. Considering this information, it is predicted that most DNA methylation changes observed here would be related to the regulation of enhancer activity since DMRs were mostly located at distal intergenic regions. The present study shows that maternal environment or stereotypies during gestation differentially affect genomic regions in piglets’ brains in relation to gene function, with introns being more prominently affected by maternal behaviour (i.e., stereotypy) than by maternal environment in the amygdala and hippocampus .

To better understand the distribution of significant DMRs across the pig genome, we also assessed the distance from the DMR to the nearest gene TSS since DNA methylation levels in TSSs are highly predictive of gene expression [111]. Our results show that the presence of DMR peaks near the TSS depends on the comparison and tissue analysed. While peaks at +20 Kbps were the most frequent in the frontal cortex, peaks at −10Kbp were the most frequent in the hippocampus. Interestingly, the only contrasts in which the amygdala showed DMRs near TSS were those involving piglets from mothers expressing stereotypy or not. Additionally, in this scenario, the frontal cortex presents no peaks. These results further support the idea that the brain regions of piglets are differentially affected by maternal environment or stereotypies. The DMRs found in the hippocampus and frontal cortex emerged mainly in relation to maternal environment, being mostly located upstream the TSS in the hippocampus and downstream the TSS in the frontal cortex. In the piglets’ amygdala, in turn, peaks of DMRs nearby TSS are not visible in relation to maternal environment, but only in relation to maternal stereotypy, supporting again the idea that the amygdala is more affected in this scenario. Future research should investigate if these epigenetic differences in genetic function affect piglets’ behaviour.

Out of all the DMRs identified, we selected 16 top-DMRs among all contrasts and tissues, which we considered to be of high relevance for exploring their biological functions. Sixteen annotated genes were identified associated with these top-DMRs, out of which nine are known genes (Supplementary Spreadsheet S3). The top-DMRs were located mostly in distal intergenic regions and introns in these genes, with only one top-DMR associated with a promoter region.

The top-DMRs located in distal intergenic regions were associated with the known genes NRN1 and U6. Interestingly, U6 in addition to presenting a distal DMR emerging in the C-E/BN contrast also exhibited an intronic DMR emerging in the H-E/BS contrast. Both NRN1 and U6 have previously been described to be involved in neuroplasticity [112–114] and cognitive function [112,113]. *NRN1*, in particular, is associated with depressive symptoms and its activity is modulated by the gene *BDNF*, which is involved in neuroplasticity [113]. *NRN1* polymorphisms are known risk factors for schizophrenia [115,116], bipolar disorders [116], and Alzheimer’s disease [117]. U6, in turn, is a non-coding small nuclear RNA (snRNA) that comprises the U6 small nuclear ribonucleoprotein (snRNP) and combines with other snRNPs and proteins to assemble the spliceosome [118]. Thus, alterations in U6 snRNA can lead to effects on the excision of introns from pre-mRNAs. Importantly, U6 is the most highly conserved of all five snRNAs comprising the spliceosome [119]. Additionally, U6 has a role in the regulation of methyl donor S-adenosylmethionine, a methyl donor for virtually all cellular methylation reactions [120].

In addition to U6, the other genes with top-DMRs in intronic regions were *FAAP24*, *FES*, *STKLD1*, *TRMT61B*, *VSIR*, and *VWA8*. FES is a proto-oncogene with cytoplasmic protein-tyrosine kinase function involved in the chemotaxis of endothelial cells [121]. Although no information exists for the role of FES in the brain, endothelial chemotaxis is required across tissues for biological processes such as embryonic development, wound healing, tissue regeneration, and tumour growth [122]. *FAAP 24* has appeared in three GWAS in relation to autism, bipolar disorder with retinitis pigmentosa, and myeloid leukaemia [123]. *STKLD1* is a serine/threonine kinase like domain for which the only information available relative to the brain is that its level of expression there only seconds that of its expression in testis, among 27 tissues analyzed in humans [124]. *TRMT61B* is the first ever described tRNA that methylates the mitochondrial 16S rRNA in all vertebrates [125]. *TRMT61B* is shown to be involved in RNA modifications in many tissues that are related to a variety of complex diseases [126] and is differentially expressed in astrocytes in relation to Alzheimer’s disease [127]. *VSIR* is a negative immune checkpoint regulator [128], and as such, with promising use in cancer treatment [129]. However, recently, immunotherapy blocking the effects of negative checkpoint regulators have been shown to affect the central nervous system [129]. VSIR, in particular, is known to be expressed in microglia and endothelial cells of the central nervous system, with its expression being differentially regulated in ageing, neuroinflammation, and diseases of the central nervous system such as neurodegeneration [130]. GWAS have linked the human *VWA8* to neurological pathologies such as autism, bipolar disorder, and comorbid migraine [130]. The only top-DMR present in a promoter region was associated with the *RCC1 gene*. RCC1 binds to chromatin and regulates chromatin condensation, having a critical role in the spindle assembly and the spatial coordination of mitosis [131]. Mutations affecting the *RCC1* domain have been associated with retinitis pigmentosa, amyotrophic lateral sclerosis, and cancer [132]. Interestingly, in glioblastoma (a cancer cell type that contains stem cells involved in therapy resistance) a molecule (EPZ020411) that inhibits PRMT6-mediated arginine methylation of the RCC1 protein prevents its stabilization, thereby improving the cytotoxic activity of radiotherapy in these tumours [131].

Next, we investigated DMR-related genes that were common between contrasts and tissues. In a similar pattern to DMRs, the hippocampus had the highest number of unique DMR-related genes (*N* = 162) across all contrasts, followed by the frontal cortex (*N* = 107) and amygdala (*N* = 102). This, combined with our findings based on the DMRs, suggests that the hippocampus is the most affected brain region by the maternal exposures investigated here in terms of not only DNA methylation changes but specifically in relation to methylation changes with gene expression consequences. Four DMR-related genes appeared in contrasts performed in all three tissues investigated: *LRATD2* (LRAT domain containing 2; Chr4), *MROH9* (maestro heat-like repeat family member 9; Chr9), *NRN1* (neuritin 1; Chr7), and U6. *LRATD2* (also known as *FAM84B*) codes for the centromeric border protein FAM84B that has oncogenic properties [133] and has been shown to be one of the eight genes considered to be major drivers of neuroendocrine carcinoma [134]. *MROH9* is one of the members of a new gene family called maestro heat-like repeat (*MROH*) for which very little information is available regarding protein structure and function, which is suspected to be mainly reproductive [135]. Thus, the role of this gene in brain tissue is completely unknown. The roles of *NRN1* and *U6* have been described above. Importantly, in relation to these two genes our results show that i) the distal intergenic region to *NRN1* containing DMRs (chr7: 3,628,367–3,628,578) had nine DMRs in common across the three brain tissues (across the different contrasts), and ii) *U6* appeared in almost all the contrasts (except A-BS/BN), with overlapping DMRs emerging from 16 (out of 24) different overlap tests performed among the contrasts.

Finally, we investigated the pathways enriched by DMR-related genes to shed light on biological processes involved in the effects of different prenatal conditions on piglets. Clusters of biological processes enriched by DMR-related genes are observed in relation to each contrast. According to the main pathways enriched in terms of genes involved and significance (Figure 7), the following are the summarized effects:

- the frontal cortex of piglets is affected in relation to neural crest development due to maternal environment; The neural crest is an important signalling centre for brain development, which regulates the secretion of the important signalling molecule FGF8 by the brain organizers isthmus and the anterior neural ridge (ANR) [136]. The ANR is then involved in specifying, via FGF8, positional identity in the neocortex [137]. Our results might imply that early developmental alterations in the neural crest are maintained and observable in the frontal cortex of piglets after birth.
- the hippocampus of piglets is affected in relation to alcohol metabolism due to both maternal environment and maternal stereotypies, and is also affected in relation to lipid mediated signalling due to both maternal environment and stereotypy; In rats, gestational protein restriction is shown to affect lipid metabolism in the brain and liver, particularly by reducing brain DHA content [138;]
- the amygdala of piglets is affected in relation to microtubule poly/depolymerization due to maternal environment, and in relation to amyloid metabolic processes due to maternal environment and maternal expression of stereotypies; microtubule poly/depolymerization affects fundamental processes in neuroplasticity, such as memory formation and learning, especially in the dendritic spines [139,140].

Although these abovementioned effects are hypothesis that need to be tested empirically in the future, our data support the idea that the maternal environment or expression of stereotypies differentially affects brain regions in the offspring in terms of biological pathways. A limitation of our study is our ability to fully separate the effects of maternal stereotypies from the effects of a barren maternal environment, as they are tightly connected. However, despite this, there are still brain epigenetic differences emerging between piglets born from mothers reared in a barren environment exhibiting and not exhibiting stereotypy. Although these are very few, some of them are unique and represent epigenetic effects specifically related to maternal stereotypy. Future research could help to further disentangle these effects in order to understand the specific contributions of each factor, maternal barren environment or stereotypy, to the offspring’s phenotype. Additionally, future research could investigate the effects of maternal environment specifically in relation to neural crest development in the frontal cortex of the offspring.

Taken together, our results provide a starting point for understanding the outcomes of maternal environment and stereotypies in the neural development, epigenome, and function of the offspring. Although the frontal cortex has previously been described as being involved in the expression of stereotypy in both pigs [141,142] and humans [143], little is known about the hippocampus and amygdala. Our combined results show that while the epigenome of the hippocampus and frontal cortex of piglets is mainly affected by the maternal environment, the epigenome of the amygdala is mainly affected by maternal stereotypies. Additionally, we showed that the molecular pathways and mechanisms triggered in the brains of piglets by maternal environment and stereotypic behaviour are also different. Future research will need to investigate whether the neuro-epigenetic alterations observed here in the brains of piglets in response to maternal environment or stereotypic behaviour would trigger concordant behavioural and neurophysiological patterns. Our study offers novel possibilities to explore the domestic pig as a model for human psychiatric disorders.

## Supplementary Material

Supplemental Material

## Data Availability

The dataset supporting the conclusions of this article is available from the European Nucleotide Archive (ENA) repository (EMBL-EBI), under the accession number PRJEB51504 (www.ebi.ac.uk/ena/data/view/PRJEB51504).
