# Supplementary material for: An enriched maternal environment and stereotypies of sows differentially affect the neuro-epigenome of brain regions related to emotionality in their piglets
Source: Epigenetics. 2023 May 16;18(1):2196656. doi: 10.1080/15592294.2023.2196656 (PMC10190189; doi:10.1080/15592294.2023.2196656)
Supplement: Supplemental Material [file KEPI_A_2196656_SM2126.zip › Supplementary files/Supplementary Tab S1.pdf]

Samples	Treatment	Depth	bp Sequenced	Breadth (bp Sequenced/depth)	% of susScr11.1
PigletsBrain18	AE	65,3316	709857131	10865448,4	0,44
PigletsBrain26	AE	66,596	685877959	10299086,4	0,42
PigletsBrain29	AE	69,8366	780145952	11171018,5	0,45
PigletsBrain47	AE	67,5788	608535349	9004826,2	0,36
PigletsBrain54	AE	58,2457	433056761	7434999,7	0,30
PigletsBrain21	AN	69,48	553256060	7962810,3	0,32
PigletsBrain32	AN	55,1685	541423433	9813995,9	0,40
PigletsBrain35	AN	60,6324	310584001	5122409,8	0,21
PigletsBrain50	AN	62,8712	659981439	10497357,1	0,42
PigletsBrain03	AT	71,0068	815525799	11485178,9	0,46
PigletsBrain06	AT	55,2693	738322196	13358631,2	0,54
PigletsBrain09	AT	65,1688	925343229	14199175,5	0,57
PigletsBrain12	AT	64,7894	674492543	10410538,5	0,42
PigletsBrain15	AT	73,2163	715423131	9771364,2	0,39
PigletsBrain38	AT	56,5033	712210143	12604753,0	0,51
PigletsBrain41	AT	64,3917	506743079	7869695,6	0,32
PigletsBrain42	AT	61,5481	569749348	9256977,0	0,37
PigletsBrain44	AT	57,6252	412785039	7163273,0	0,29
PigletsBrain53	AT	51,5093	429749836	8343150,4	0,34
PigletsBrain19	CE	75,6951	722560897	9545676,0	0,39
PigletsBrain24	CE	76,0081	705626917	9283575,3	0,37
PigletsBrain27	CE	77,6505	624221398	8038858,7	0,32
PigletsBrain30	CE	74,6424	1108664458	14853011,9	0,60
PigletsBrain48	CE	39,3202	230698498	5867175,1	0,24
PigletsBrain22	CN	70,264	672149635	9566059,9	0,39
PigletsBrain33	CN	70,8521	644093035	9090669,6	0,37
PigletsBrain36	CN	75,5293	578735124	7662392,3	0,31
PigletsBrain51	CN	75,8236	650672224	8581394,5	0,35
PigletsBrain01	CT	66,0554	696819970	10549023,5	0,43
PigletsBrain04	CT	66,8334	665193231	9953006,0	0,40
PigletsBrain07	CT	63,8921	1197976175	18749989,0	0,76
PigletsBrain10	CT	66,5533	800509089	12028090,1	0,49
PigletsBrain13	CT	79,8399	984409957	12329799,5	0,50
PigletsBrain16	CT	61,6252	743038978	12057388,5	0,49
PigletsBrain39	CT	66,8636	555176341	8303117,7	0,34
PigletsBrain43	CT	71,6217	643873595	8989923,4	0,36
PigletsBrain45	CT	73,1208	579220674	7921421,5	0,32
PigletsBrain20	HE	68,2734	894677793	13104339,2	0,53
PigletsBrain25	HE	80,3432	746070507	9286044,2	0,37
PigletsBrain28	HE	81,6823	578877234	7086936,0	0,29
PigletsBrain31	HE	74,1346	768945550	10372289,7	0,42
PigletsBrain49	HE	66,614	612173319	9189859,8	0,37
PigletsBrain23	HN	73,8267	527685353	7147622,1	0,29
PigletsBrain34	HN	63,0848	730510053	11579810,9	0,47
PigletsBrain37	HN	69,438	559836284	8062390,7	0,33
PigletsBrain52	HN	63,3937	562861515	8878824,2	0,36
PigletsBrain02	HT	65,5786	1126592745	17179274,1	0,69
PigletsBrain05	HT	62,7254	1272807856	20291745,5	0,82

PigletsBrain08	HT	65,9684	713024921	10808582,9	0,44
PigletsBrain11	HT	68,853	711584949	10334843,1	0,42
PigletsBrain14	HT	69,676	845236438	12130955,3	0,49
PigletsBrain17	HT	73,1956	1722346568	23530739,1	0,95
PigletsBrain40	HT	64,8514	482432884	7439051,2	0,30
PigletsBrain46	HT	52,442	507138937	9670472,8	0,39
**TotalAverage**		66,9	702768620,9	10483315,61	0,42
**TotalSD**		7,9	243988369	3418962	0,14

Based on: **Sscrofa11.1, INSDC Assembly GCA_000003025.6, Dec 2016**

Per Treatment

**Treatment**	**Depth ±SD**			**Number of bp Sequenced±SD**				**Breadth (bp Sequenced/depth) ±SD**			**% of the Sus.scrofa genome v.11.1 Covered ±SD**		
**AE**	65,52	±	4,39	643494630	±	132646297	±	9755076	±	1539431	0,39	±	0,06
**AN**	62,04	±	5,92	516311233	±	147151130	±	8349143	±	2402893	0,34	±	0,10
**AT**	62,10	±	6,95	650034434	±	167168391	±	10446274	±	2404062	0,42	±	0,10
**CE**	68,66	±	16,44	678354434	±	312726294	±	9517659	±	3317923	0,38	±	0,13
**CN**	73,12	±	2,97	636412505	±	40274694	±	8725129	±	814627	0,35	±	0,03
**CT**	68,49	±	5,52	762913112	±	207706079	±	11209084	±	3273055	0,45	±	0,13
**HE**	74,21	±	6,83	720148881	±	127602691	±	9807894	±	2193342	0,40	±	0,09
**HN**	67,44	±	5,17	595223301	±	91584944	±	8917162	±	1910765	0,36	±	0,08
**HT**	65,41	±	6,17	922645662	±	425044924	±	13923208	±	5724438	0,56	±	0,23
**Total**	67,44	±	4,27	680615355	±	118263338	±	10072292	±	1403430	0,41	±	0,06
