# Supplementary material for: An enriched maternal environment and stereotypies of sows differentially affect the neuro-epigenome of brain regions related to emotionality in their piglets
Source: Epigenetics. 2023 May 16;18(1):2196656. doi: 10.1080/15592294.2023.2196656 (PMC10190189; doi:10.1080/15592294.2023.2196656)
Supplement: Supplemental Material [file KEPI_A_2196656_SM2126.zip › Supplementary files/Supplementary Tab S2.pdf]

Gene	Contrast	Role	Diseases-related	Full description
**NRN1**	Amygdala: hypermethylated in the distal intergenic region of the barren environment group, in relation to the enriched environment.	Neuroplasticity	Schizophrenia, Alzheimer, bipolar disorders.	*NRN1* is related to neuronal structures associated with plasticity and promotes neuronal migration (Zito et al., 2014; Bosserhoff et al., 2017). *NRN1* gene is associated with depressive symptoms, and in such cases is modulated by the gene BDNF, involved in neuroplasticity (Prats et al., 2017). The variability of *NRN1* is a shared risk factor for schizophrenia (Chandler et al., 2010; Fatjó-Vilas et al., 2015), bipolar disorders (Fatjó-Vilas et al., 2015), and Alzheimer's diseases (Piras et al., 2019). Furthermore, *NRN1* has a role as modifier of the cognitive functioning in schizophrenia (Chandler et al., 2010). Neurotrophic factors are essential to the well-functioning of the brain, and its disruption is related to the reduction of plasticity, which in turns, could underlie the variability in cognitive functioning related to the development of mental disorders (Bosserhoff et al., 2017; Prats et al., 2017).
**RCC1**	Frontal cortex: hypermethylated in the frontal cortex of piglets from sows that expressed stereotypic behavior, when compared with piglets from sows that did not expressed.	Cell cycle, cell signalling, membrane trafficking.	Retinitis pigmentosa, amyotrophic lateral sclerosis, and cancer.	The gene *RCC1* is a regulator of chromosome condensation 1 (Mashimo et al., 2009). Proteins with HECT domains have been described to function as ubiquitin ligases, and those that contain *RCC1*-like domains have been reported to functions as GTPases regulators (Mashimo et al., 2009). These two activities are essential in a number of important cellular processes such as cell cycle, cell signaling, and membrane trafficking (Mashimo et al., 2009). Mutations affecting these domains have been found associated with retinitis pigmentosa, amyotrophic lateral sclerosis, and cancer (Mashimo et al., 2009).
**FES**	Frontal cortex: hypermethylated in the enriched environment group, in relation to the group expressing stereotypies.	Signaling downstream of the receptors for multiple cytokines, growth factors, immunoglobin receptors, and innate immune system.	Acute myelogenous leukemia growth cell.	This is one of the genes implicated in signaling downstream of the receptors for multiple cytokines, growth factors, immunoglobin receptors, and innate immune system (see review Greer, 2002). The FES gene is a protein-tyrosine kinase-encoding retroviral oncogene. This gene has been described with the involvement in different types of cancer, including the contrasting oncogenic and tumor suppressor roles, that are intrinsic to cancer cells (Greer et al., 2012). The inhibition of FES might be sufficient to block acute myelogenous leukemia growth cell (Weir et al., 2017), the most common hematologic malignancy in adults (Estey and Dohner, 2006).

**U6**	Frontal cortex: hypermethylated in the group not expressing stereotypies, when compared with the group from environmental enrichment. Also described in the intron region, hypermethylated in the hippocampus in the group from enriched environment, in relation to the group expressing stereotypies.	Methyl donor S-adenosylmethionine.		One of the functions of *U6* is considered conserved in vertebrates and evolved to regulate methyl donor S-adenosylmethionine (Pendleton et al., 2017). S-adenosylmethionine is considered as methyl donor for nearly all cellular methylation reactions (Pendleton et al., 2017).
**STKLD1**	Hippocampus: hypermethylated in the enriched environment group, when contrasted with the group not expressing stereotypies, in the intron region.	Transferase activity, transferring phosphorus-containing groups and protein tyrosine kinase activity; protein kinases also control many other cellular processes, including metabolism, transcription, cell cycle progression, cytoskeletal rearrangement and cell movement, apoptosis, and differentiation.		The gene *STKLD1* is related to transferase activity, transferring phosphorus-containing groups and protein tyrosine kinase activity. Protein kinases mediate most of the signal transduction in eukaryotic cells, by modification of substrate activity, protein kinases also control many other cellular processes, including metabolism, transcription, cell cycle progression, cytoskeletal rearrangement and cell movement, apoptosis, and differentiation (Manning et al., 2002).

**TRMT61B**	Hippocampus: hypermethylated in the intron region, in the animals from enriched environment in the prenatal period when compared with not expressing stereotypies group.	Mitochondrial 16S rRNA is methylated by tRNA methyltransferase TRMT61B in all vertebrates, revealing a conserved mechanism of rRNA modification that has been selected instead of DNA mutations to enable proper mitochondrial ribosome function.	Alzheimer	Differential expression of *TRMT61B* in astrocytes was associated with Alzheimer's (Sekar et al., 2015; Bar-Yaacov et al., 2016). Moreover, mitochondrial 16S rRNA is methylated by tRNA methyltransferase *TRMT61B* in all vertebrates, revealing a conserved mechanism of rRNA modification that has been selected instead of DNA mutations to enable proper mitochondrial ribosome function (Sekar et al., 2015; Bar-Yaacov et al., 2016). The astrocytes have an important cellular and molecular role to the biological functioning of the brain, including neurovascular communication and immune function (Nuriya and Hirase, 2016).
**FAAP24**	Hippocampus: hypermethylated in the enriched group, when compared with the group expressing stereotypies.	Genomic integrity.	Related to the Fanconi anemia.	The type of the gene *FAAP24* is protein coding, and has been demonstrated to play dual roles in DNA damage response against crosslinking lesions (Wang et al., 2013a). Regarding disease, *FAAP 24* is related to the Fanconi anemia, a disorder associated with failure in the DNA repair (Coulthard et al., 2013). *FAAP24* is also related in human patients with Epstein Barr virus associated lymphoproliferation in case of loss of function mutation (Daschkey et al., 2016). In combination with *FANCM*, the gene *FAAP24* plays several roles in the genomic integrity, although both do not overlap in their functions (Wang et al., 2013b).

**VWA8**	Hippocampus: hypermethylated in the enriched group, compared with barren environment.	ATPase activity in mitochondria, and it is presence exclusively in mitochondria raises the possibility that this protein has a role in metabolic regulation or bioenergetics events.	Neurological and oncological pathologies, which include autism, bipolar disorder, comorbid migraine, and acute myeloid leukemia.	*VWA8* is associated with ATPase activity in mitochondria, and it is presence exclusively in mitochondria raises the possibility that this protein has a role in metabolic regulation or bioenergetics events (Luo et al., 2017). Genome-wide associated studies have linked human *VWA8* to neurological and oncological pathologies, which include autism, bipolar disorder, comorbid migraine, and acute myeloid leukemia (Grewe et al., 2018). High methylation and low expression of this gene is linked with better outcomes in acute myeloid leukemia (Marcucci et al., 2014). This gene also appears as a possible signal in three genome wide association studies, for serum calcium concentrations, autism, bipolar disorder with retinitis pigmentosa, and myeloid leukemia (Luo et al., 2017). The hypermethylation of the gene in the intragenic region, may be associated with its repression, but not always.
**VSIR**	Hippocampus: hypermethylated in the intron region of the DMR in the enriched group, when contrasting with the group not expressing stereotypies.	Negative immune checkpoint regulator.	Malignant mesothelioma, a highly lethal cancer; immunohistochemical diagnostic marker for epithelioid mesothelioma.	*VSIR* is a type protein coding gene, recognized as a negative immune checkpoint regulator, reported to be expressed in malignant mesothelioma, a highly lethal cancer (Hmeljak et al., 2018). *VSIR* compounds the B7 family of negative checkpoints regulator. Moreover, *VSIR* expression may be used as an immunohistochemical diagnostic marker for epithelioid mesothelioma (Chung et al., 2019).
