# Supplementary figures and images for: An enriched maternal environment and stereotypies of sows differentially affect the neuro-epigenome of brain regions related to emotionality in their piglets
Source: Epigenetics. 2023 May 16;18(1):2196656. doi: 10.1080/15592294.2023.2196656 (PMC10190189; doi:10.1080/15592294.2023.2196656)

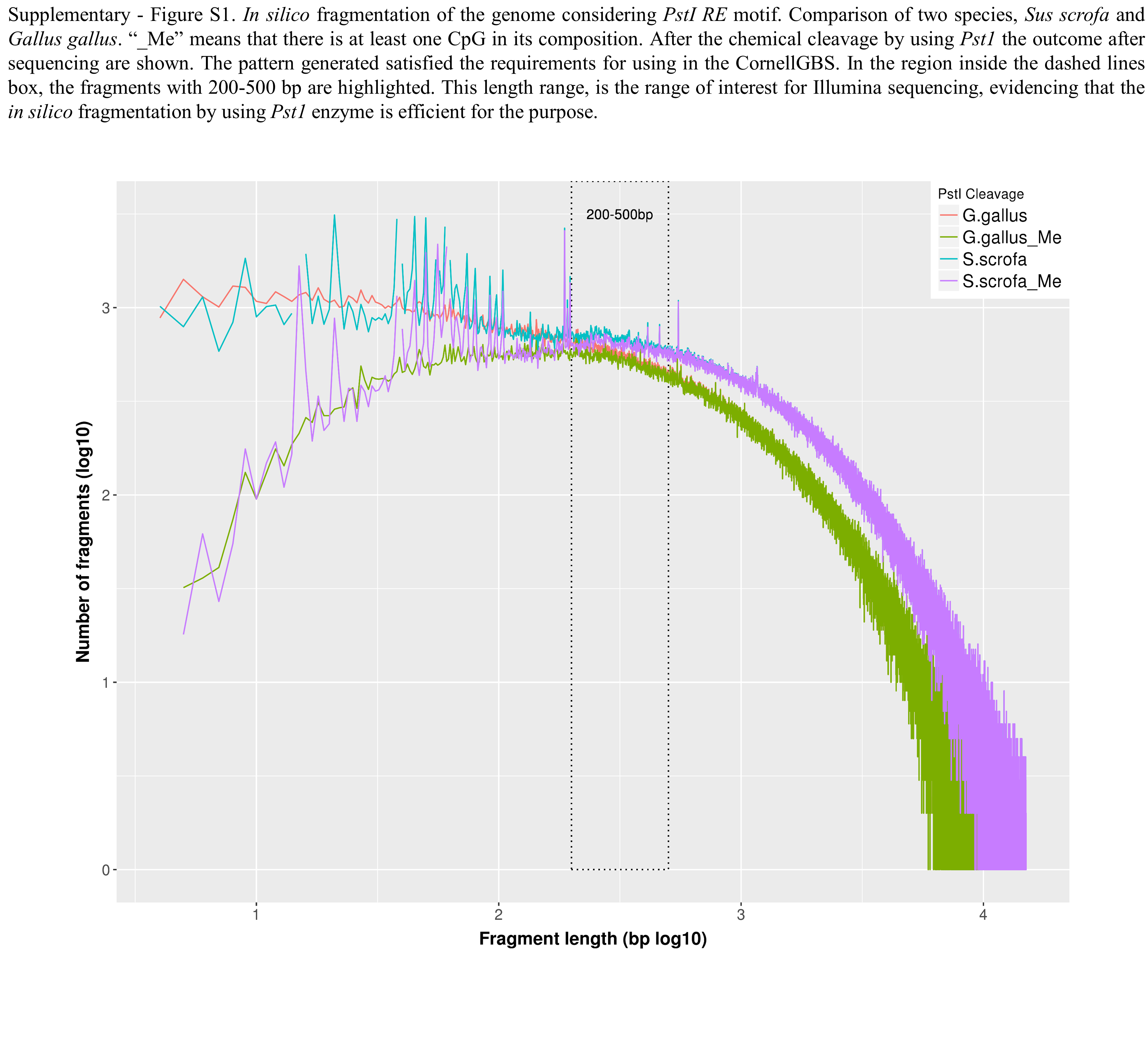

Supplement: Supplemental Material [file KEPI_A_2196656_SM2126.zip › Supplementary files/Supplementary Figure S1.TIF]

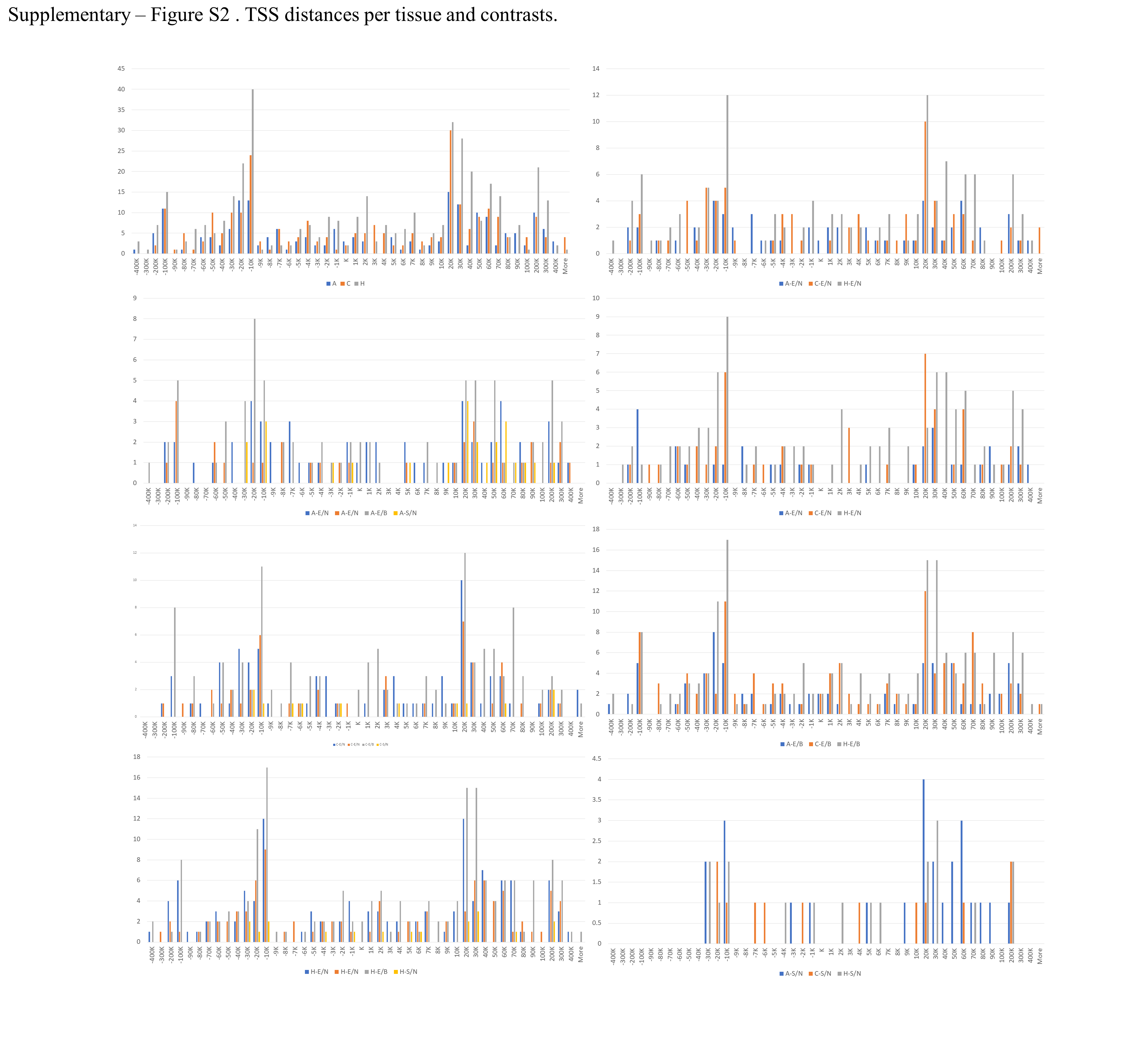

Supplement: Supplemental Material [file KEPI_A_2196656_SM2126.zip › Supplementary files/Supplementary Figure S2.TIF]
